# Operationalizing health equity: a mixed-methods approach to measuring organizational capacity in public health practice

**DOI:** 10.3389/fpubh.2026.1913380

**Published:** 2026-09-30

**Authors:** Stephanie Mazzucca-Ragan, Rebekah R. Jacob, Douglas A. Luke, Sara Malone, Cheryl A. Valko, Ross C. Brownson

**Affiliations:** 1 Prevention Research Center, Bursky School of Public Health, Washington University in St. Louis, St. Louis, MO, United States; 2 Bursky School of Public Health, Washington University in St. Louis, St. Louis, MO, United States; 3 Alvin J. Siteman Cancer Center, Washington University School of Medicine, Washington University in St. Louis, St. Louis, MO, United States

**Keywords:** concept mapping, confirmatory factor analysis, health equity, organizational capacity, psychometric evaluation, public health practice

## Abstract

**Introduction:**

Health equity is a core function of public health practice, yet most public health agencies lack tools to assess their internal capacity to advance it. Existing measurement efforts have focused primarily on community-level health disparities rather than the organizational infrastructure, leadership, and practices needed to address them. This study aimed to develop a reliable survey for measuring organizational equity capacity within public health agencies.

**Methods:**

We used a two-phase, mixed-methods approach. First, we identified priority organizational domains through concept mapping with 73 state and local practitioners. Findings informed the development of a 47-item survey administered to a national sample of practitioners (*n* = 234). We conducted psychometric analyses and confirmatory factor analysis (CFA) to refine the instrument and evaluate model fit.

**Results:**

Concept mapping identified eight domains: Funding, Agency Practices, Communication, Data Practices, Organizational Partnerships, Community Representation, Leadership, and Staff Development. Through CFA, we identified a 32-item, eight-factor survey with strong model fit (CFI = 0.908; RMSEA = 0.066). All subscale domains showed good to excellent internal consistency (Cronbach's alpha = 0.79–0.91).

**Conclusion:**

Organizational health equity capacity is multidimensional rather than a single undifferentiated construct. This pragmatic and reliable 32-item instrument provides a rigorous tool for agencies to assess internal equity infrastructure. Agencies can use this tool to benchmark organizational capacity and track systemic progress toward institutionalizing health equity.

## Introduction

Health equity is a core function for public health practice (1–3). The imperative of a health equity approach is driven in part by decades of rising income inequality (4), the visibility and impacts of historic structural racism (5), and the need to view public health as a common good rather than a commodity (6). It involves moving beyond simply measuring health disparities to ensuring every individual has a fair and just opportunity to be healthy (7). An aim of advancing health equity is making health a shared societal responsibility. The concept is evident in national, state, and local strategic plans, as well as in the notion of Public Health 3.0, where local leaders are “public health strategists” who influence the social determinants of health through partnerships across multiple sectors (e.g., education, transportation, justice, housing) (1).

To support progress tracking, public health agencies are guided by the axiom: “what gets measured, gets done (8).” Many public health agencies support their measurement function using surveillance systems for certain health conditions or risk factors (e.g., disease registries, ongoing surveys of behavioral risk factors, hospital discharge data). Additionally, agencies have traditionally invested resources in developing and implementing monitoring systems for community and health outcomes. These data can be supplemented with various indices of social deprivation (e.g., area deprivation index, social deprivation index). Several public health systems [e.g., the King County determinants of equity data tool (9)] have developed equity-relevant datasets (e.g., on education, economic development, employment, and criminal justice) (10).

Despite its importance, there are limited data on the extent to which public health agencies are comprehensively addressing health equity. A broader data ecosystem is needed to measure not only community-level indicators but also awareness, skills, leadership, and performance within public health agencies and work units. In a 2016 nationwide survey of US practitioners in state health departments, only 2% reported working primarily on health equity, and 9% reported that health equity was one of their multiple priority areas (11). National data collected in 2017 showed that more than half of public health agency staff believe their agencies should be very involved in improving health equity, yet few reported being very involved in addressing specific social determinants of health (e.g., transportation, housing) (12). In a more recent (2022) study on health equity practices among state public health practitioners (13), the strongest areas were the staff's ability to describe the causes of health inequities (82% agreeing or strongly agreeing) and to identify disparities within their jurisdictions (82%). Only 32% of respondents reported having systems in place to track progress on health equity.

Efforts to measure organizational, rather than community, capacity for equity are not new, though they remain fragmented. A scoping review of indicators available to Ontario local public health agencies found few that were evidence-based or validated, and concluded that those worth retaining needed substantial further definition before use (14). Guidance developed during the COVID-19 pandemic took a different route, appending health equity prompts to an existing set of public health emergency preparedness indicators so that agencies would articulate equity considerations at each stage of organizational decision-making (15). Most recently, a scoping review identified 17 organizational health equity capacity assessments for public health organizations and found limited evidence on how these tools had been implemented and what they yielded (16). The present study advances this literature in three respects. First, practitioners generated the domains rather than deriving them from expert consensus or document review. Second, the resulting items were subjected to confirmatory factor analysis in a national sample, yielding evidence of internal structure and reliability. Third, the instrument measures capacity at the work unit level, where staff time, data, and partnerships are largely allocated.

The overall goal of the current study was to develop a new set of health equity measures for public health practice, using best practices in measurement development (17–21). We accomplished our goal with three activities, in which we: (1) conducted concept mapping (22) to identify priority health equity domains, (2) developed a survey instrument, with cognitive testing, to measure these core domains, and (3) conducted a national survey of public health practitioners to perform confirmatory factor analysis and assess the measurement structure of the instrument.

## Methods

This study was approved by the Washington University in St. Louis Institutional Review Board (IRB# 202506181).

### Mixed-methods participatory input

Concept mapping is a mixed-methods, participatory approach used to gather and organize ideas through several steps: develop the focus prompt; identify participants; generate ideas; structure ideas; analyze data and create visual representations; and interpret and use results (23). GroupWisdom, a web-based concept mapping software, was used throughout the process.

#### Statement generation (brainstorming)

Statement generation was conducted in December 2024. Participants included program staff and leadership from state and local public health departments. Contact lists were obtained from our organizational partners, i.e., the Big Cities Health Coalition, the Missouri Public Health Institute, the National Association of Chronic Disease Directors, and the de Beaumont Foundation. A total of 315 individuals were invited to participate. After removing invalid email addresses, bounce-backs, and individuals no longer in their positions, 295 were eligible.

Participants accepted a written consent statement in the software and were then asked to complete the focus prompt with as many statements as they desired. Participants responded to the prompt: “*For a public health agency to advance health equity, it needs…”* All previously generated statements were visible to participants, and statements were anonymous. A total of 201 unique statements were generated. The research team reviewed the statements to remove redundancies and consolidate conceptually similar responses. Through iterative discussion and consensus, the list was reduced to 82 unique statements, which were carried forward to the sorting and rating steps. The statement list was finalized in alignment with concept mapping best practices, which recommend limiting the sorting and rating phases to fewer than 100 unique statements (24).

#### Sorting and rating

Sorting and rating were conducted between January and February 2025. A total of 299 individuals (eligible from Phase 1 and additional contacts that were recommended by participants) were invited to participate; 290 were deemed eligible after invalid or ineligible contacts were removed. Participants sorted the 82 statements into groups that they identified as conceptually meaningful and assigned a name to each group. They could create as many or as few groupings as they wanted. Each statement could appear in only one pile, and no statement could be sorted into its own separate pile. Following this task, participants rated each statement separately on two dimensions, importance and feasibility, using a 7-point Likert scale (1 = low; 7 = high). Importance and feasibility ratings were used to characterize participants' views of each statement and were not used to select or prioritize domains. All eight clusters were carried forward into instrument development.

#### Cluster analysis and map solution

The GroupWisdom platform organizes the sorted data using several quantitative methods applied sequentially. A similarity matrix is created first to consolidate the sorting data from all participants. Then, multidimensional scaling is used to create a point map in which each statement is represented as a point on a two-dimensional plane (25, 26). Distances between points reflect how often statements were sorted together; points that are closer together are interpreted as more conceptually similar. Hierarchical cluster analysis is used to group points representing similar concepts (27, 28). Of the 56 participants who completed the rating activities, 53 produced valid sorts, meaning the participant placed every statement into a group, created more than one group but fewer groups than the total number of statements, and did not place any statement in a group by itself. All 53 valid sorts were included in the cluster analysis; all 56 participants were included in the importance and feasibility analyses. The research team examined cluster maps of four to 15 cluster solutions to determine which best reflected the point map and were most interpretable. Each cluster map was evaluated for the consistency of ideas and using the statistics reported by GroupWisdom. The statistics evaluated were the multidimensional scaling stress value, which indexes how closely the two-dimensional point map reproduces the original sorting data; bridging values for each statement, which indicate whether a statement was sorted consistently with the statements nearest to it on the map (values near 0) or bridged separate regions of the map (values near 1); and the number of statements assigned to each cluster across candidate solutions. Cluster solutions that split conceptually cohesive groups or produced clusters too broad to label were rejected.

Ratings of each statement were averaged, and an overall cluster average was calculated. A ladder graph was produced in GroupWisdom to visualize and compare the cluster-level averages for the importance and feasibility ratings. Additionally, a “Go-Zone” map was created that assigns each statement to one of the two ratings, creating a priority-quadrant plot to guide future efforts to advance health equity in public health practice.

Based on coherence and interpretability of the clusters, the research team selected the eight-cluster solution as the best-fitting model. Following member checking with participants, minor refinements were made to cluster labels and item assignments, in accordance with standard concept mapping practices. The final eight clusters were: (1) Funding (5 items); (2) Agency Practices (11 items); (3) Communication (7 items); (4) Data Practices (16 items); (5) Organizational Partnerships (10 items); (6) Community Representation (14 items); (7) Leadership (7 items); and 8) Staff Development (12 items). These clusters informed the development of the subsequent survey instrument.

### Instrument development and psychometric assessment

#### Instrument development

Using best practices for item development, the survey items were derived directly from the concept mapping results and from two previous surveys (11, 13, 21). The research team met to review the concept mapping statements and ratings and to develop an initial set of survey items that best reflected the priority areas in each domain. Statements were edited according to measurement development processes, for example, removing double-barreled items. Following development of the pilot tool, the team conducted cognitive testing of the survey with public health professionals (*N* = 6) to assess clarity, relevance, and interpretability (29). One round of revisions took place to incorporate cognitive interview feedback prior to administering the survey.

The initial instrument included 47 items organized into eight sections (five to seven items per section) corresponding to the concept mapping clusters. All 47 initial items, and the model in which each removed item was dropped, are listed in Table S2. The start of the instrument included the directions: “*Please indicate how often each of the following activities has occurred at your work unit over the past 12 months.”* Response options were measured on a 6-point Likert scale (1 = Almost never; 6 = Almost always) with an option for “Not able to answer”. Five or more statements per section were selected at this stage to allow for removal of poorly performing items after initial testing, using the criteria described below.

#### Pilot sample and data collection

A random sample of 1,416 individuals was drawn from our partner organizations' membership list and individuals who participated in the concept mapping process, to ensure representation of practitioners and partners with expertise in chronic disease prevention and familiarity with the equity-related constructs being measured. An additional 12 individuals were added through self-nomination or colleague recommendations to capture relevant expertise not reflected in the membership list, bringing the total to 1,428 invitations distributed via email in August 2025.

Five reminder emails were sent to non-respondents over an eight-week period. The survey closed in October 2025. After removing invalid email addresses and automated replies indicating extended leave or separation from the agency, 1,295 individuals were deemed eligible. Of those, 234 completed the survey, yielding an 18% response rate. Respondents represented 53 U.S. states and territories.

#### Analyses and model fit evaluation

Data were managed and analyzed in R. We conducted descriptive analyses, visualization, and psychometric analyses. We used the psych package in R to calculate measures of internal consistency (reliability), including Cronbach's alpha (30).

Confirmatory factor analysis was conducted to assess the fit of the proposed eight-factor model using the lavaan R package (31). The base model was specified with one factor and all 47 items from the survey using a robust maximum likelihood (MLR) estimator and full information maximum likelihood (FIML) to handle missing data. Responses of “Not able to answer” were recoded as missing and handled under FIML alongside item nonresponse. Missing responses accounted for 3.7% of all item responses. Items were considered poorly performing and were candidates for removal if they cross-loaded onto other factors based on modification indices, e.g., when the highest modification indices included one item and factors in which the item was not originally placed. Additionally, items that were highly correlated with another item (>0.7) were considered for removal; in such cases, the item that had the stronger factor loading was retained.

Model fit was evaluated using multiple indices, including the chi-square test of model fit, Comparative Fit Index (CFI), Tucker-Lewis Index (TLI), Root Mean Square Error of Approximation (RMSEA), and Standardized Root Mean Square Residual (SRMR). Although a non-significant chi-square test (*p* > 0.05) indicates good model fit, this test is highly sensitive to large sample sizes. Therefore, additional fit indices were emphasized. CFI and TLI values range from 0 to 1, with values ≥0.90 indicating acceptable fit and ≥0.95 indicating good fit. RMSEA values < 0.05 indicate close fit, values between 0.05 and 0.08 indicate reasonable fit, and values ≥0.10 indicate poor fit (32, 33). Confidence intervals around RMSEA were examined to assess approximate model fit. SRMR values were also evaluated, with thresholds similar to RMSEA and less sensitivity to sample size.

## Results

### Mixed-methods participatory input

#### Participants

Seventy-three participants completed the statement generation activity (25% response rate). Fifty-six participants completed the rating tasks (19% response rate), and 53 of these produced valid sorts. Across the concept mapping steps, participants represented 35 U.S. states and six U.S. territories. In the brainstorming step, 56% of respondents were from local health departments and 33% from state health departments (Table 1). Similarly, in the sorting and rating steps, 54% were from local and 39% from state health departments. Participants had substantial professional experience, with a mean of 13.5 years (SD = 10.2) and 12.7 years (SD = 8.7) in their current role for the brainstorming and sorting/rating steps, respectively.

**Table 1:** Participant characteristics, health equity in public health practice, United States, 2025.

Characteristic	Concept mapping phase 1 (*N* = 73)	Concept mapping phase 2 (*N* = 56)
Organization		
Local health department	41 (56%)	30 (54%)
State health department	24 (33%)	22 (39%)
Other categories with *N* = 3 or less	8 (11%)	4 (7%)
Years in their role (mean, SD)	13.5 (10.2)	12.7 (8.7)
Role		
Program manager or coordinator	18 (25%)	17 (31%)
Department head	10 (14%)	10 (19%)
Epidemiologist	7 (10%)	6 (11%)
Health equity/health disparities coordinator	6 (8%)	4 (7%)
Health educator	3 (4%)	2 (4%)
Other categories with *N* = 2 or less	29 (39%)	15 (28%)
Equity in their work^*^		
A small portion (1–24%)	7 (9%)	7 (13%)
Some portion (25–49%)	14 (19%)	11 (20%)
A significant portion (50–74%)	26 (36%)	14 (25%)
Nearly all (75–100%)	26 (36%)	24 (43%)
Identify as member of underrepresented population in research^**^		
Yes	31 (42%)	25 (45%)
No	34 (47%)	27 (48%)
Prefer not to respond	8 (11%)	4 (7%)

^*^In your current position, what proportion of your overall work addresses health equity and/or health disparities? ^**^Do you identify as a member of a historically underrepresented population in research by race, ethnicity, disability, sexual orientation, gender identity, socioeconomic status, or other aspects of identity?

Respondents represented a range of professional roles. In the brainstorming step, the most common roles were program manager/coordinator (25%), department head (14%), and epidemiologist (10%). Participants who sorted and rated were similarly distributed, with program managers/coordinators (31%) and department heads (19%) most frequently represented. More than 70% of participants reported that at least half of their work addressed health equity or health disparities, and approximately half identified as members of a historically underrepresented population in research.

#### Concept map

Sorting analyses yielded an eight-cluster solution that was retained based on interpretability and statistical fit (stress index = 0.228). The stress index is a goodness-of-fit statistic produced by multidimensional scaling that quantifies the discrepancy between the two-dimensional point map and participants' original sorting data. It ranges from 0 to 1, and lower values indicate that the map represents the sorting data more faithfully. In a pooled analysis of 69 concept mapping studies, stress values averaged 0.28 and ranged from 0.17 to 0.34; the value observed here falls below that average, indicating good fit (24). The final clusters were (1) Funding, (2) Agency Practices, (3) Communication, (4) Data Practices, (5) Organizational Partnerships, (6) Community Representation, (7) Leadership, and (8) Staff Development (Figure 1, Table S1).

**Figure 1:**
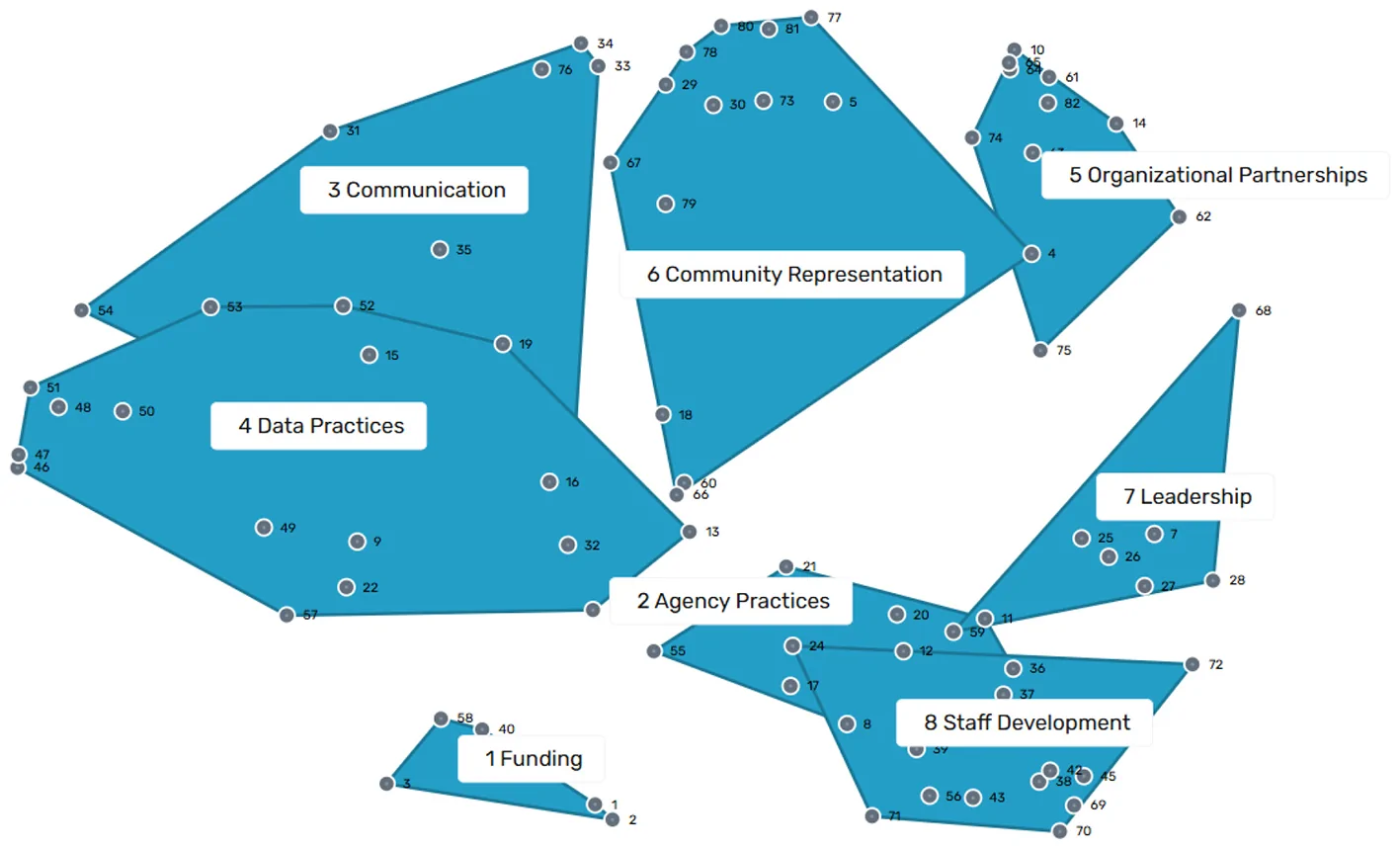
Cluster map, health equity in public health practice.

#### Importance and feasibility ratings

Across clusters, statements were generally rated highly for importance (means largely ≥5.4 on a 7-point scale), while feasibility ratings were more variable (Figure S1). Several statements fell into the high-importance/high-feasibility quadrant (Quadrant I; Figure S2), including *fostering collaboration across sectors* and *incorporating social determinants of health into equity efforts*. In contrast, several items were rated highly important but less feasible (Quadrant IV), including *ensuring sufficient funding and fostering political leadership at multiple levels*. Overall, participants consistently rated *structural, systems-level, and partnership-oriented strategies* as highly important, with feasibility varying depending on resource and political considerations (Figures S1, S2).

### Instrument development

#### Descriptive statistics and internal consistency

A total of 234 respondents completed the survey (18% response rate). Respondents represented 53 U.S. states and territories. The initial 47-item survey was organized into eight factors aligned with the concept mapping clusters (Table S2).

#### Confirmatory factor analysis

The eight-factor structure derived from the concept mapping exercise was supported by the pilot data. Model fit indices are presented in Table 2. The baseline one-factor model demonstrated poor fit (CFI = 0.701; TLI = 0.687; RMSEA = 0.096; SRMR = 0.072). The hypothesized eight-factor full model improved fit (CFI = 0.826; TLI = 0.813; RMSEA = 0.074; SRMR = 0.067), though indices remained below established thresholds for good fit.

**Table 2:** Summary of model fit indices, health equity in public health practice, United States, 2025.

Fit index	Single-factor model 47 items, 1 factor	Initial eight-factor model 47 items, 8 factors	Reduced eight-factor model 40 items, 8 factors	Final eight-factor model 32 items, 8 factors
*p*-value for χ^2^	0.000	0.000	0.000	0.000
CFI	0.701	0.826	0.866	0.908
TLI	0.687	0.813	0.853	0.896
RMSEA	0.096	0.074	0.071	0.066
SRMR	0.072	0.067	0.062	0.051

CFI, Comparative Fit Index; TLI, Tucker-Lewis Index; RMSEA, Root Mean Square Error of Approximation; SRMR, Standardized Root Mean Square Residual.

Two reduced models were tested. The first, a reduced model (40 items) further improved model fit (CFI = 0.866; TLI = 0.853; RMSEA = 0.071; SRMR = 0.062). The final model (32 items) demonstrated the best overall fit, meeting or approaching recommended thresholds (CFI = 0.908; TLI = 0.896; RMSEA = 0.066; SRMR = 0.051). Although chi-square statistics were statistically significant across all models, this was expected given sample size sensitivity.

### Final survey domains, items, and measurement properties

The eight-factor, 32-item model was retained as the final survey measure, as it demonstrated the best balance of statistical fit and conceptual relevance (Table 3). Conceptual relevance was evident in that all eight domains identified through concept mapping were retained in the final model, each represented by four items, so that item reduction did not eliminate any practitioner-identified domain. The retained items also preserved the substantive content of each domain. Leadership, for example, retained items covering accountability, systemic change, and political navigation, and Data Practices retained items covering progress tracking, evaluation, presentation of data to varied audiences, and attention to specific subgroups. Mean item scores ranged from 3.73 to 5.49 on a 6-point scale, indicating moderate to high frequency of reported activities.

**Table 3:** Survey domains and items, health equity in public health practice.

Item	Mean	SD
Domain 1: Agency practices (Cronbach's alpha: 0.85)	5.27	0.77
My work unit maintains health disparity reduction as a core value	5.49	0.84
My work unit adapts programs for populations experiencing disparities	5.29	0.87
My work unit ensures I can prioritize under-resourced and marginalized communities	5.21	0.94
My work unit intentionally incorporates disparity reduction in most policies and operations	5.10	1.00
Domain 2. Communication (Cronbach's alpha: 0.79)	4.84	0.81
My work unit ensures transparent external communication to establish community trust	5.02	1.01
My work unit captures the voices of those with lived experience to address disparities	4.61	1.05
My work unit represents all populations in visual communications	4.96	0.98
My work unit disseminates data on specific subgroups to community members	4.79	1.11
Domain 3. Data practices (Cronbach's alpha: 0.85)	4.91	0.90
My work unit has systems to track our progress in addressing health disparities	4.70	1.26
My work unit evaluates plans to address health disparities	4.90	1.04
My work unit tailors data presentations to reach various audiences	4.97	1.02
My work unit considers multiple groups to address disparities	5.06	0.98
Domain 4. Organizational partnerships (Cronbach's alpha: 0.80)	4.66	0.90
My work unit fosters collaboration with governmental sectors outside of health to address health disparities	4.76	1.19
My work unit establishes collaborations between public health practice and academic institutions to address health disparities	4.77	1.08
My work unit strengthens partnerships between public health and nonprofit organizations to address health disparities	5.05	0.99
My work unit strengthens partnerships between public health and businesses to address health disparities	4.05	1.31
Domain 5. Staff development (Cronbach's alpha: 0.89)	4.63	1.04
My work unit ensures that existing staff have health training to understand the underlying causes of disparities	4.69	1.22
My work unit provides staff training in the collection, interpretation, and use of health disparities data	4.34	1.31
My work unit hires individuals who know about health disparity reduction	4.60	1.18
My work unit engages staff in health disparity efforts	4.90	1.08
Domain 6. Leadership (Cronbach's alpha: 0.91)	4.41	1.23
Leaders in my work unit are held accountable for addressing disparities	4.36	1.39
Leaders in my work unit embrace systemic change when addressing health disparities	4.71	1.25
Leaders in my work unit are willing to take on health disparities despite potential political fallout	4.28	1.43
Leaders in my work unit can negotiate political challenges when addressing health disparities	4.14	1.41
Domain 7. Community representation (Cronbach's alpha: 0.90)	4.34	1.06
My work unit shares decision-making authority with partners when addressing disparities	4.55	1.08
My work unit co-creates solutions with those most impacted by disparities	4.11	1.24
My work unit ensures communities are involved in planning and implementation to address disparities	4.34	1.22
My work unit utilizes community voices when developing strategic plans for health programs	4.39	1.22
Domain 8. Funding (Cronbach's alpha: 0.80)	4.19	1.10
My work unit maintains sufficient funding to support disparity-related priorities	4.06	1.35
My work unit funds external partners to support innovative programs that decrease health disparities	4.35	1.47
My work unit shares resources across categorical grants that address specific diseases or risk factors	4.54	1.28
My work unit uses economic evaluation data in its decision-making related to disparities	3.73	1.40

“Not able to answer” responses varied across domains. In the final instrument, these responses were least common for Organizational Partnerships (0.9%) and Agency Practices (1.2%), and most common for Funding (6.2%) and Leadership (9.4%); the three items drawing the most such responses all belonged to these two domains. Sixty-seven percent of respondents answered every item in the final instrument.

Domain-level means, calculated for each respondent as the mean of their available items within a domain, ranked as follows: Agency Practices (5.27), Data Practices (4.91), Communication (4.84), Organizational Partnerships (4.66), Staff Development (4.63), Leadership (4.41), Community Representation (4.34), and Funding (4.19).

This ranking follows a consistent pattern. Domains describing internally controlled, values-oriented activity scored highest and varied least across respondents: every item in Agency Practices had a mean at or above 5.10 and a standard deviation below 1.00, the narrowest spread of any domain. Domains that depend on resources, political conditions, or the redistribution of authority scored lowest and varied most. Funding, Leadership, and Community Representation each contained items with standard deviations above 1.20, and four of the five lowest-scoring items in the instrument fall within these domains: using economic evaluation data in decision-making (M = 3.73), maintaining sufficient funding for disparity-related priorities (M = 4.06), co-creating solutions with those most impacted by disparities (M = 4.11), and negotiating political challenges (M = 4.14). The fifth, partnerships with businesses (M = 4.05), is described below.

Variation within domains was also informative. Organizational Partnerships spanned the widest range of any domain, from partnerships with nonprofit organizations (M = 5.05) to partnerships with businesses (M = 4.05), suggesting that agencies partner readily within the nonprofit and governmental sectors but engage the private sector far less often. In Community Representation, sharing decision-making authority with partners (M = 4.55) scored higher than co-creating solutions with those most impacted by disparities (M = 4.11). In Leadership, the item on embracing systemic change (M = 4.71) scored higher than the two items involving political risk (M = 4.28 and M = 4.14). Across the instrument, agencies reported doing the work they control more often than the work that requires resources, political cover, or direct involvement of affected communities.

Inter-factor correlations ranged from 0.567 to 0.868 (Table S3). The strongest correlation was observed between Communication and Community Representation (r = 0.868), followed by Data Practices and Staff Development (r = 0.825). Leadership was strongly correlated with Staff Development (r = 0.800) and Community Representation (r = 0.776). Funding demonstrated moderate correlations with all other domains (r range: 0.635–0.680). Organizational Partnerships was the most distinct domain in the model. It had the lowest average correlation with the other seven domains (mean r = 0.65) and the single lowest inter-factor correlation in the matrix, with Leadership (r = 0.567); its correlations with Staff Development (r = 0.607) and Agency Practices (r = 0.613) were also among the lowest observed. This pattern indicates that an agency's external partnership activity is not a simple function of its internal equity infrastructure, and that the two can develop independently. While correlations were substantial, they remained below 0.90, indicating related but distinct constructs.

## Discussion

Integrating stakeholder-driven concept mapping with rigorous psychometric testing, this study establishes a multidimensional measure of foundational characteristics that define a public health agency's capacity to systematically advance health equity. Eight factors were identified through a mixed-methods approach, spanning agencies' internal operations and their integration of community members and organizational partners who are integral to advancing health equity in their communities. This study provides one of the first empirically derived tools to assess equity capacity at the work unit level and moves the field from a conceptual discussion of equity toward operational indicators of capacity. Importantly, this work highlights that advancing health equity requires systems-level organizational change, not isolated programmatic efforts.

Our findings provide empirical evidence that public health practitioners view health equity capacity as a multidimensional organizational system. Rather than focusing on individual programs or isolated policies designed to reduce health disparities, the resulting map suggests that advancing equity requires a complex integration of skills, leadership commitment, and infrastructure woven across a public health agency, similar to other frameworks that align health equity work with the core functions of governmental public health (3). Further, this organizational system does not operate in a vacuum but requires collaboration between public health agencies, their organizational partners, and members of the communities they serve. The focus on external partners in our concept map aligns with Public Health 3.0, which reframes public health practitioners as conveners of cross-sector partnerships and community members and organizations to advance population health (1). By defining these interconnected domains and developing items that measure each domain, our findings move the field toward a concrete, operational roadmap for institutionalizing health equity within the day-to-day functions of public health agencies.

The domains our participants generated correspond to dimensions identified in prior reviews of organizational health equity capacity, including leadership commitment, workforce development, resource allocation, and data infrastructure (16). Where our results depart from that work is in how finely practitioners distinguish the internal conditions supporting equity. In developing indicators for Ontario agencies, expert informants found that items concerning organizational systems and support for equity work did not fit the four groupings used to organize the indicator set (assessing and reporting on health status, modifying programs for priority populations, engaging in multisectoral collaboration, and policy advocacy), prompting the addition of a fifth category encompassing organizational and system development (14). Cohen et al.'s framework of organizational capacity for public health equity action characterizes an organization's internal context through three dimensions: shared values, commitment and will, and organizational infrastructure (34). The examples they give of infrastructure include access to and ability to interpret local data on inequities, advocacy skills among the workforce, and processes ensuring that equity-seeking populations can influence organizational decision-making. These three examples correspond to Data Practices, Staff Development, and Community Representation in our instrument, each of which our analysis supports as a distinct factor. Practitioners appear to treat these internally facing dimensions as separate concepts, which matters for measurement because an agency may be well staffed yet without the data to target its efforts, or data-rich yet unable to bring affected communities into decisions about how that data is used.

The survey results highlight a stark disconnect between high-level organizational values and the operational realities of public health practice. While respondents reported that their units overwhelmingly maintain disparity reduction as a core value (M = 5.49), factors capturing the concrete execution of equity initiatives received substantially lower ratings. Specifically, agencies reported significant difficulty navigating political challenges (M = 4.14), securing dedicated funding (M = 4.06), and authentically co-creating solutions with the communities most impacted by disparities (M = 4.11). This gap suggests that while administrative endorsement is well-established, the field lacks the structural infrastructure necessary to bring affected communities into decision-making and withstand external political pressures (13). These internal capacity deficits are exacerbated by systemic workforce pressures: a lack of demographic representativeness hinders authentic community engagement (35), while the high financial burden of graduate education limits the entry of diverse talent and undermines the sustainability of equity-focused staff development (36). While these broader systemic barriers are beyond the control of any single agency, providing a psychometrically sound method for tracking agency capacity represents a necessary step toward organizational accountability.

The development of this instrument is strengthened by a mixed-methods design that integrated practitioner-driven concept mapping with quantitative validation, ensuring the domains and survey items are both grounded in practice and psychometrically sound. Also, survey respondents represented 53 states and territories, indicating that these concepts are relevant nationwide. Further, the high internal consistency of all eight factors (Cronbach's alphas between 0.79 and 0.91) suggests that the items within each domain are conceptually cohesive and clearly understood by practitioners as distinct organizational constructs.

However, these findings should be considered alongside some limitations. While our sample was geographically and professionally diverse, the modest response rate introduces the potential for nonresponse bias. Because the sampling frame drew on membership lists of organizations focused on chronic disease prevention and equity, and because more than 70% of respondents reported doing equity-related work or belonging to an underrepresented group, respondents were likely more engaged with equity work than the broader public health workforce. Estimates of organizational capacity reported here may reflect an upper bound rather than an average. Additionally, using a self-report format may introduce social desirability bias; however, practitioners' perceptions are the most appropriate metric for these domains. These internal perceptions are the primary drivers of organizational decision-making, the allocation of staff time, and the ultimate development and sustainment of structures that support equity. If social desirability influenced responses, its effect likely varied across domains. Agency Practices, which asks respondents to affirm that their unit holds disparity reduction as a core value and incorporates it into policies and operations, is the domain tied most closely to stated organizational values, and it showed the highest mean and the smallest variance of the eight domains. Domains asking about observable resource and authority conditions, including Funding, Leadership, and Community Representation, showed lower means and substantially wider spread. This is the pattern that would be expected if items closer to organizational values drew more uniformly favorable responses than items closer to operational reality, and it echoes the gap between endorsement of health equity and reported involvement in specific activities found in earlier national surveys (12, 13). The same pattern is also consistent, however, with genuine differences in how difficult these domains are to enact. Distinguishing between the two will require validating the instrument against external indicators such as budget allocations, staffing records, and documented community agreements.

Our measure provides a standardized method for tracking organizational equity characteristics over time. Internally, agencies can use the eight-factor survey to track their own capacity. The concept mapping ratings reported here reflect the views of 56 practitioners at one point in time and should not be treated as a general prioritization scheme; agencies should prioritize based on their own staff and partners' perceptions of importance and feasibility. Standardizing these metrics also provides a rigorous, data-driven foundation for the 'Public Health Capacity Teams' proposed by Bishai and colleagues, who advocate for multisectoral, participatory evaluations of both internal infrastructure and the external capabilities required to operationalize equity in the Public Health 3.0 era (37). Future research should strengthen the tool's psychometric properties by testing predictive validity for community outcomes, sensitivity to change, and performance across diverse agency types. Finally, exploring the tool's utility in restrictive political contexts remains vital.

Comparing the ratings with the survey data shows what practitioners considered important and achievable alongside what their units do, and the two diverge most clearly within Organizational Partnerships. Six of the ten partnership statements fell into the high-importance/high-feasibility quadrant, while none of the Funding or Leadership statements did (Table S1). Practitioners rated partnering with other agencies, fostering collaboration with sectors outside health, and committing to consistent engagement with partners as both important and achievable. Yet the corresponding survey items received only moderate scores (domain mean 4.66), and the domain correlated least strongly with the rest of the instrument. Partnership work is therefore neither contested nor viewed as out of reach, and yet it is not consistently occurring. That combination makes Organizational Partnerships a distinct target for near-term action, and distinguishes it from Funding and Leadership, where low scores accompany low perceived feasibility and the barriers lie largely outside any single agency. Agencies looking for tractable starting points can prioritize partnership development, and particularly partnerships with businesses, which was both the lowest-scoring partnership item (M = 4.05) and an area practitioners rated as feasible. Treating partnership development as a standing element of program planning, rather than a project-specific activity, would convert an area of high perceived feasibility into measurable practice.

The pattern of “Not able to answer” responses points the same direction. Although these responses were infrequent, they differed across domains. Practitioners most often selected “Not able to answer” for items about their unit's leadership and funding conditions, and least often for items about its partnership activity. The domains that scored lowest and were rated least feasible are also the ones respondents most often declined to rate, which is consistent with those conditions being set above the work unit rather than within it. We could not test this directly, since position level was not captured in the pilot survey. The sampling frame suggests most respondents were middle managers and frontline program staff rather than agency executives, which would place funding and leadership decisions above the level at which they work. Capturing position level in future administrations would test this explanation and would also show who within an agency is best positioned to assess which domains.

## Conclusion

This study demonstrates that practitioners view equity capacity as a multidimensional organizational system rather than isolated activities. This reliable eight-factor instrument provides a rigorous tool for agencies to assess and strengthen the internal infrastructure required to operationalize health equity. Advancing equity is an ongoing process requiring sustained structural investment, measurable capacity, and the political will to navigate systemic challenges. As practices must adapt to shifting political and funding landscapes, these domains represent a dynamic framework that should evolve with the field. Future efforts should prioritize longitudinal application of this tool to track institutional progress and drive the systemic transformations necessary to improve population health.

## Data Availability

The raw data supporting the conclusions of this article will be made available by the authors, without undue reservation.
