# Supplementary material for: Operationalizing health equity: a mixed-methods approach to measuring organizational capacity in public health practice
Source: Front Public Health. 2026 Sep 30;14:1913380. doi: 10.3389/fpubh.2026.1913380 (PMC13661554; doi:10.3389/fpubh.2026.1913380)
Supplement: Supplementary file 1 [file Table_1.DOCX]

**Supplemental File**

**Table S1.** Concept Mapping Items by Domain, Health equity in public health practice, United States, 2025

| **Item Number** | **Item** | **Importance – Mean** | **Importance – SD** | **Feasibility – Mean** | **Feasibility – SD** | **Priority Quadrant**^a^ |
| --- | --- | --- | --- | --- | --- | --- |
| **Funding** | | | | | | |
| 1 | Ensure sufficient funding to support equity-related priorities. | 6.11 | 1.11 | 3.57 | 1.54 | IV:HI-LF |
| 2 | Allow flexibility in work with less rigid grants (especially CDC categorical grants). | 5.52 | 1.34 | 3.57 | 1.67 | III: LI-LF |
| 3 | Provide funding to support innovative programs that decrease health disparities. | 5.87 | 1.06 | 4.02 | 1.60 | IV:HI-LF |
| 40 | Evaluate the impact of the agency on health equity. | 5.66 | 1.21 | 5.28 | 1.27 | II: LI-HF |
| 58 | Be held to realistic expectations for public health programs (e.g., we cannot solve equity with our CDC grant funds and yet are asked to address equity without the CDC making any changes to reduce systemic oppression). | 5.78 | 1.18 | 4.61 | 1.53 | III: LI-LF |
| **Agency Practices** | | | | | | |
| 8 | Become more expert at facilitating processes and activities at the systems level. | 5.43 | 1.27 | 5.33 | 1.24 | II: LI-HF |
| 11 | Incorporate equity intentionally in all policies and operations. | 6.04 | 1.12 | 5.06 | 1.39 | IV:HI-LF |
| 12 | Acknowledge that our agencies may be perpetuating inequities. | 5.72 | 1.41 | 5.09 | 1.56 | II: LI-HF |
| 17 | Work toward a genuine dismantling of white supremacy culture around public health. | 5.43 | 1.80 | 3.63 | 1.83 | III: LI-LF |
| 20 | Commit to centering racial justice in all health equity work. | 5.56 | 1.48 | 4.72 | 1.56 | III: LI-LF |
| 21 | Acknowledge the role of racism and all the other forms of discrimination (e.g., sexism, ageism, ableism) that prevent people from getting necessary care and resources. | 5.83 | 1.37 | 5.28 | 1.47 | I: HI-HF |
| 23 | Create a common organizational understanding of what "advance health equity" means (e.g., to reduce health disparities in the community, operate as an agency more equitably internally and externally). | 5.89 | 0.90 | 5.70 | 1.30 | I: HI-HF |
| 36 | Commit to creating and sustaining an internal culture of diversity, equity, and inclusion to ensure authentic approaches (i.e., lead by example). | 6.06 | 1.34 | 5.24 | 1.54 | I: HI-HF |
| 37 | Complete honest internal reviews of the health agency's own culture and operational practices that impact equity. | 5.85 | 1.02 | 5.07 | 1.43 | I: HI-HF |
| 39 | Demonstrate equity within the agency. | 5.91 | 1.07 | 5.26 | 1.18 | I: HI-HF |
| 55 | Prioritize equity in each program's planning, development, and evaluation. | 6.04 | 1.13 | 5.48 | 1.30 | I: HI-HF |
| **Communication** | | | | | | |
| 31 | Use social media effectively without causing alarm or distrust in populations experiencing inequities. | 5.44 | 1.19 | 5.61 | 1.31 | II: LI-HF |
| 32 | Determine a less politicized way of talking about health equity. | 5.41 | 1.55 | 4.76 | 1.44 | III: LI-LF |
| 33 | Ensure transparent communication to establish trust throughout communities. | 6.22 | 0.98 | 5.56 | 1.16 | I: HI-HF |
| 34 | Listen and learn from community members through storytelling. | 5.81 | 1.18 | 5.91 | 0.98 | I: HI-HF |
| 35 | Represent all populations in visual communications (e.g., representing a diverse array of skin tones in photo galleries). | 5.74 | 1.15 | 5.81 | 1.33 | II: LI-HF |
| 54 | Disseminate disaggregated data to community members. | 5.43 | 1.33 | 5.28 | 1.42 | II: LI-HF |
| 76 | Capture the voice of those with lived expertise and have the intention to work together on what's learned. | 6.19 | 0.93 | 5.70 | 1.08 | I: HI-HF |
| **Data Practices** | | | | | | |
| 6 | Understand how policies impact health and work to address those. | 5.96 | 0.95 | 5.78 | 0.98 | I: HI-HF |
| 9 | Apply a comprehensive, systems-based approach that addresses the root causes of health disparities to promote fairness in access to health services, resources, and opportunities. | 6.04 | 1.06 | 4.81 | 1.35 | IV:HI-LF |
| 13 | Address systemic barriers to promote social determinants of health. | 6.13 | 1.12 | 4.61 | 1.37 | IV:HI-LF |
| 15 | Assess the root causes of health disparities in a community (e.g., healthcare access, nutritious food access, cultural impacts, stigma) | 6.39 | 0.88 | 5.54 | 1.14 | I: HI-HF |
| 16 | Promote understanding that health outcomes are often determined by an individual's environment, which includes access to resources. | 6.07 | 0.95 | 5.80 | 1.09 | I: HI-HF |
| 19 | In equity efforts, consider multiple identity groups (e.g., race, ability, sexual orientation, gender, country of national origin, language). | 6.13 | 1.05 | 5.69 | 1.21 | I: HI-HF |
| 22 | Allow the necessary time to see results regarding health equity efforts (i.e., addressing the hundreds of years of racial injustice and knowing change will take time). | 5.61 | 1.20 | 4.57 | 1.60 | III: LI-LF |
| 46 | Obtain better data systems and make data modernization a priority of equity efforts. | 5.63 | 1.14 | 4.69 | 1.31 | III: LI-LF |
| 47 | Advocate for and participate in equitable data collection. | 5.89 | 0.95 | 5.41 | 1.11 | I: HI-HF |
| 48 | Assemble quantitative and qualitative data from all relevant groups, including conducting ethnographic studies on hard-to-reach populations. | 5.74 | 1.23 | 4.76 | 1.32 | III: LI-LF |
| 49 | Move past "we need more data" to actions tied to clear goals and objectives. | 5.44 | 1.27 | 4.94 | 1.38 | III: LI-LF |
| 50 | Identify health outcomes in all geographical areas of the jurisdiction. | 5.87 | 1.10 | 5.33 | 1.32 | I: HI-HF |
| 51 | Understand how to tailor data to reach a variety of audiences. | 5.91 | 0.98 | 5.85 | 0.90 | I: HI-HF |
| 52 | Build a comprehensive understanding of communities being served (e.g., demographics, current health status, resources available to residents). | 6.24 | 0.85 | 5.87 | 1.12 | I: HI-HF |
| 53 | Assess community needs in health equity without blind spots. | 5.96 | 0.95 | 5.04 | 1.41 | IV:HI-LF |
| 57 | Design and evaluate a plan to address health equity. | 5.79 | 1.17 | 5.46 | 1.49 | I: HI-HF |
| **Organizational Partnerships** | | | | | | |
| 10 | Support political advocacy among partners. | 5.17 | 1.13 | 4.13 | 1.43 | III: LI-LF |
| 14 | Foster active cooperation by policymakers and community partners to remove obstacles to equitable health. | 5.94 | 1.04 | 4.39 | 1.48 | IV:HI-LF |
| 61 | Partner with other agencies (e.g., organizations, hospitals, clinics, health systems) to advance health equity. | 6.26 | 1.03 | 5.76 | 0.93 | I: HI-HF |
| 62 | Obtain support from local agency governing bodies to address health equity. | 5.67 | 0.99 | 4.54 | 1.33 | III: LI-LF |
| 63 | Establish collaborations between public health practice and universities to address health equity. | 5.26 | 1.23 | 5.46 | 1.21 | II: LI-HF |
| 64 | Foster stronger partnerships between public health, nonprofits, and local businesses. | 6.19 | 0.91 | 5.76 | 0.97 | I: HI-HF |
| 65 | Foster collaboration with sectors outside of health (e.g., transportation, education, housing). | 6.33 | 0.89 | 5.74 | 1.14 | I: HI-HF |
| 74 | Commit to consistent engagement with partners and the populations served. | 6.31 | 0.80 | 5.70 | 1.11 | I: HI-HF |
| 75 | Have a willingness to work collaboratively with humility. | 6.15 | 1.04 | 6.06 | 0.98 | I: HI-HF |
| 82 | Develop meaningful relationships with other government, non-profit, and for-profit organizations to focus on upstream factors. | 6.28 | 0.98 | 5.60 | 1.08 | I: HI-HF |
| **Community Representation** | | | | | | |
| 4 | Share power when focusing on upstream causes (e.g., income inequality, racism) impacting inequities. | 5.81 | 1.29 | 4.44 | 1.63 | IV:HI-LF |
| 5 | Co-create solutions with those most impacted by inequities. | 6.25 | 0.94 | 5.41 | 1.17 | I: HI-HF |
| 18 | Incorporate social determinants of health into health equity efforts (e.g., affordable housing, transportation, built environment, education, food security). | 6.44 | 0.77 | 5.41 | 1.37 | I: HI-HF |
| 29 | Seek community input and participation, even when uncomfortable for agency leaders and staff. | 6.46 | 0.82 | 5.44 | 1.31 | I: HI-HF |
| 30 | Ensure rural communities are involved in health equity planning and implementation. | 5.91 | 1.34 | 5.30 | 1.28 | I: HI-HF |
| 60 | Recognize that one size does not fit all in equity efforts. | 5.96 | 1.05 | 5.87 | 1.12 | I: HI-HF |
| 66 | Do not deny anyone health care treatment for any reason. | 5.78 | 1.61 | 4.43 | 2.12 | III: LI-LF |
| 67 | Ensure all community members have equal access to all resources and information to optimize health. | 5.87 | 1.29 | 4.55 | 1.64 | IV:HI-LF |
| 73 | Include contributing voices from the social sciences (e.g., anthropology, sociology), whether from staff or community members. | 5.17 | 1.22 | 5.04 | 1.15 | III: LI-LF |
| 77 | Involve neighborhood associations to generate participation in health equity conversations. | 5.50 | 1.24 | 5.06 | 1.37 | III: LI-LF |
| 78 | Utilize community voice when developing strategic plans for health programs. | 6.15 | 0.94 | 5.44 | 1.16 | I: HI-HF |
| 79 | Address immediate concerns of communities while also working towards systemic changes for long-term solutions. | 6.07 | 0.89 | 4.93 | 1.21 | IV:HI-LF |
| 80 | Pay community members for their time and contributions in a relevant way (i.e., more than a gift card, with benefits such as childcare or transportation). | 5.54 | 1.44 | 4.04 | 1.80 | III: LI-LF |
| 81 | Engage representative grassroots groups from every relevant geographic level to identify local needs and solutions. | 5.89 | 1.04 | 5.11 | 1.34 | I: HI-HF |
| **Leadership** | | | | | | |
| 7 | Have a champion willing to embrace systemic change. | 5.41 | 1.27 | 4.65 | 1.51 | III: LI-LF |
| 25 | Obtain buy-in and support from leadership to be fully committed to advancing health equity. | 6.07 | 1.04 | 4.76 | 1.34 | IV:HI-LF |
| 26 | Ensure that agency leaders reflect the communities they serve. | 5.35 | 1.31 | 4.24 | 1.49 | III: LI-LF |
| 27 | Hold leaders accountable for addressing health equity. | 5.81 | 1.23 | 4.28 | 1.46 | IV:HI-LF |
| 28 | Foster leaders willing to take on health equity despite potential political fallout. | 5.61 | 1.50 | 3.96 | 1.69 | III: LI-LF |
| 59 | Be accountable for actions to address inequities. | 5.74 | 1.14 | 4.89 | 1.41 | III: LI-LF |
| 68 | Foster political leadership at national, state, and local levels to support health equity. | 5.80 | 1.19 | 3.85 | 1.70 | IV:HI-LF |
| **Staff Development** | | | | | | |
| 24 | Understand the difference between health disparities and health equity. | 5.65 | 1.38 | 6.39 | 0.94 | II: LI-HF |
| 38 | Provide dedicated staff to facilitate health equity work across an agency. | 5.28 | 1.46 | 4.22 | 1.50 | III: LI-LF |
| 41 | Ensure that their staff reflect the communities they serve. | 5.57 | 1.28 | 4.56 | 1.31 | III: LI-LF |
| 42 | Hire individuals who know about health equity. | 5.06 | 1.29 | 5.15 | 1.32 | II: LI-HF |
| 43 | Ensure staff have expertise in health equity, racial equity, and structural racism. | 5.46 | 1.53 | 5.17 | 1.55 | II: LI-HF |
| 44 | Obtain staff buy-in for health equity efforts. | 5.60 | 1.25 | 4.98 | 1.16 | III: LI-LF |
| 45 | Establish an equity officer who has the power to implement changes. | 4.76 | 1.55 | 3.83 | 1.54 | III: LI-LF |
| 56 | Build health equity measures into staff performance evaluations. | 5.02 | 1.41 | 4.76 | 1.70 | III: LI-LF |
| 69 | Train staff on what health equity is and what it means. | 5.74 | 1.20 | 6.15 | 1.25 | II: LI-HF |
| 70 | Offer training on cultural humility for all staff. | 5.31 | 1.27 | 5.80 | 1.38 | II: LI-HF |
| 71 | Advance staff training in health equity data, interpretation, and utilization. | 5.61 | 1.12 | 5.63 | 1.25 | II: LI-HF |
| 72 | Make health equity training of staff and organizations a core contract requirement with partner organizations. | 5.26 | 1.43 | 4.78 | 1.57 | III: LI-LF |

^a^ HI: high importance, LI: low importance, HF: high feasibility, LF: low feasibility

**Figure S1.** Ladder graph illustrating alignment between perceived importance and feasibility ratings of health equity-related domains, Health equity in public health practice, United States, 2025

**Figure S2.** Go-Zone quadrant map of the 82 concept mapping statements by mean importance and mean feasibility ratings, Health equity in public health practice, United States, 2025.

Each point represents one statement. Mean importance is plotted on the horizontal axis and mean feasibility on the vertical axis, both rated on a 7-point scale. The number beside each point is the statement number listed in Table S1, where the full text of the statement, its mean importance and feasibility ratings, and its assigned domain can be found. Quadrant boundaries are placed at the mean rating for each axis (importance = 5.78; feasibility = 5.07). Quadrant I (upper right) contains statements rated above average on both dimensions; Quadrant II (upper left), below-average importance and above-average feasibility; Quadrant III (lower left), below average on both; and Quadrant IV (lower right), above-average importance and below-average feasibility. Points are colored by domain, as shown in the legend. The correlation between mean importance and mean feasibility ratings across statements was r = 0.41.

**Table S2.** Survey Development Item-level Summary, Health Equity in Public Health Practice

| **Item** | **Mean** | **SD** | **Notes** |
| --- | --- | --- | --- |
| **Factor 1: Agency Practices** | | | |
| My work unit maintains health disparity reduction as a core value. | 5.49 | 0.84 |  |
| My work unit adapts programs for populations experiencing disparities. | 5.29 | 0.87 |  |
| My work unit ensures I can prioritize under-resourced and marginalized communities. | 5.21 | 0.94 |  |
| My work unit intentionally incorporates disparity reduction in most policies and operations. | 5.10 | 1.00 |  |
| My work unit creates and sustains an internally diverse and inclusive culture. | 4.93 | 0.95 | Dropped in reduced model |
| My work unit intentionally hires staff members who represent communities experiencing disparities. | 4.30 | 1.30 | Dropped in final model |
| **Factor 2. Communication** | | | |
| My work unit ensures transparent external communication to establish community trust. | 5.02 | 1.01 |  |
| My work unit captures the voices of those with lived experience to address disparities. | 4.61 | 1.05 |  |
| My work unit represents all populations in visual communications. | 4.96 | 0.98 |  |
| My work unit disseminates data on specific subgroups to community members. | 4.79 | 1.11 |  |
| My work unit uses a less politicized way of discussing health disparity reduction. | 5.00 | 1.05 | Dropped in reduced model |
| My work unit uses social media in ways that build trust among populations experiencing disparities. | 4.25 | 1.33 | Dropped in final model |
| **Factor 3. Data Practices** | | | |
| My work unit has systems to track our progress in addressing health disparities. | 4.70 | 1.26 |  |
| My work unit evaluates plans to address health disparities. | 4.90 | 1.04 |  |
| My work unit tailors data presentations to reach various audiences. | 4.97 | 1.02 |  |
| My work unit considers multiple groups to address disparities. | 5.06 | 0.98 |  |
| My work unit assembles both quantitative and qualitative data for program evaluation. | 5.20 | 0.97 | Dropped in reduced model |
| My work unit assesses the root causes of health disparities in a community. | 4.43 | 1.15 | Dropped in final model |
| **Factor 4. Organizational Partnerships** | | | |
| My work unit fosters collaboration with governmental sectors outside of health to address health disparities. | 4.76 | 1.19 |  |
| My work unit establishes collaborations between public health practice and academic institutions to address health disparities. | 4.77 | 1.08 |  |
| My work unit strengthens partnerships between public health and nonprofit organizations to address health disparities. | 5.05 | 0.99 |  |
| My work unit strengthens partnerships between public health and businesses to address health disparities. | 4.05 | 1.31 |  |
| My work unit has an intent to work collaboratively with community partners in a respectful and humble manner. | 5.48 | 0.86 | Dropped in reduced model |
| In my work unit, health disparities reduction is explicitly stated as a shared priority in collaborations with internal agency partners. | 4.92 | 1.20 | Dropped in final model |
| **Factor 5. Staff Development** | | | |
| My work unit ensures that existing staff have health training to understand the underlying causes of disparities. | 4.69 | 1.22 |  |
| My work unit provides staff training in the collection, interpretation, and use of health disparities data. | 4.34 | 1.31 |  |
| My work unit hires individuals who know about health disparity reduction. | 4.60 | 1.18 |  |
| My work unit engages staff in health disparity efforts. | 4.90 | 1.08 |  |
| My work unit allocates staff time to address health disparities. | 4.84 | 1.19 | Dropped in final model |
| **Factor 6. Leadership** | | | |
| Leaders in my work unit are held accountable for addressing disparities. | 4.36 | 1.39 |  |
| Leaders in my work unit embrace systemic change when addressing health disparities. | 4.71 | 1.25 |  |
| Leaders in my work unit are willing to take on health disparities despite potential political fallout. | 4.28 | 1.43 |  |
| Leaders in my work unit can negotiate political challenges when addressing health disparities. | 4.14 | 1.41 |  |
| Leaders in my work unit place a high value on addressing health disparities. | 5.26 | 1.03 | Dropped in final model |
| **Factor 7. Community Representation** | | | |
| My work unit shares decision-making authority with partners when addressing disparities. | 4.55 | 1.08 |  |
| My work unit co-creates solutions with those most impacted by disparities. | 4.11 | 1.24 |  |
| My work unit ensures communities are involved in planning and implementation to address disparities. | 4.34 | 1.22 |  |
| My work unit utilizes community voices when developing strategic plans for health programs. | 4.39 | 1.22 |  |
| My work unit prioritizes community members’ input when determining health department priorities. | 4.58 | 1.09 | Dropped in reduced model |
| My work unit incorporates root causes of health into health disparities efforts. | 4.64 | 1.12 | Dropped in reduced model |
| My work unit pays community members for their time and contributions in a meaningful way. | 3.60 | 1.70 | Dropped in final model |
| **Factor 8. Funding** | | | |
| My work unit maintains sufficient funding to support disparity-related priorities. | 4.06 | 1.35 |  |
| My work unit funds external partners to support innovative programs that decrease health disparities. | 4.35 | 1.47 |  |
| My work unit shares resources across categorical grants that address specific diseases or risk factors. | 4.54 | 1.28 |  |
| My work unit uses economic evaluation data in its decision-making related to disparities. | 3.73 | 1.40 |  |
| My work unit encourages accountability through transparent financial practices. | 4.66 | 1.33 | Dropped in reduced model |
| My work unit allocates resources based on communities’ needs and priorities to address past and present disparities. | 4.60 | 1.19 | Dropped in final model |

**Table S3.** Correlation coefficients between domains of the final instrument, Health equity in public health practice, United States, 2025

|  | **Agency Practices** | **Communication** | **Data Practices** | **Organizational Partnerships** | **Staff Development** | **Leadership** | **Community Representation** | **Funding** |
| --- | --- | --- | --- | --- | --- | --- | --- | --- |
| **Agency Practices** | 1.000 |  |  |  |  |  |  |  |
| **Communication** | 0.690 | 1.000 |  |  |  |  |  |  |
| **Data Practices** | 0.779 | 0.835 | 1.000 |  |  |  |  |  |
| **Organizational Partnerships** | 0.613 | 0.748 | 0.710 | 1.000 |  |  |  |  |
| **Staff Development** | 0.710 | 0.718 | 0.825 | 0.607 | 1.000 |  |  |  |
| **Leadership** | 0.737 | 0.751 | 0.704 | 0.567 | 0.800 | 1.000 |  |  |
| **Community Representation** | 0.659 | 0.868 | 0.666 | 0.687 | 0.675 | 0.776 | 1.000 |  |
| **Funding** | 0.680 | 0.656 | 0.641 | 0.635 | 0.663 | 0.656 | 0.676 | 1.000 |
